# Communities of Arbuscular Mycorrhizal Fungi in the Roots of *Pyrus pyrifolia* var. *culta* (Japanese Pear) in Orchards with Variable Amounts of Soil-Available Phosphorus

**DOI:** 10.1264/jsme2.ME12118

**Published:** 2012-12-19

**Authors:** Yuko Yoshimura, Akifumi Ido, Koji Iwase, Teruyuki Matsumoto, Masahide Yamato

**Affiliations:** 1 The United Graduate School of Agricultural Sciences, Tottori University, 4–101 Koyama-Minami, Tottori 680–8553, Japan; 2 Tottori Prefectural Agriculture and Forest Research Institute, Horticultural Experiment Center, Hokuei, Tohaku, Tottori 689–2221, Japan; 3 Depertment of Natural and Environmental Science, Teikyo University of Science, 2525 Yatsusawa, Uenohara 409–0193, Japan; 4 Fungus/Mushroom Resource and Research Center, Faculty of Agriculture, Tottori University, 4–101 Koyama-Minami, Tottori 680–8553, Japan

**Keywords:** AML1-AML2, principal component analysis (PCA), redundancy analysis (RDA), soil-available P, SSU rDNA

## Abstract

We examined the colonization rate and communities of arbuscular mycorrhizal fungi (AMF) in the roots of *Pyrus pyrifolia* var. *culta* (Japanese pear) in orchards to investigate the effect of phosphorus (P) fertilization on AMF. Soil cores containing the roots of Japanese pear were collected from 13 orchards in Tottori Prefecture, Japan. Soil-available P in the examined orchards was 75.7 to 1,200 mg kg^−1^, showing the extreme accumulation of soil P in many orchards. The AMF colonization rate was negatively correlated with soil-available P (*P* <0.01). AMF communities were examined on the basis of the partial fungal DNA sequences of the nuclear small-subunit ribosomal RNA gene (SSU rDNA) amplified by AMF-specific primers AML1 and AML2. The obtained AMF sequences were divided into 14 phylotypes, and the number of phylotypes (species richness) was also negatively correlated with soil-available P (*P* <0.05). It was also suggested that some AM fungi may be adapted to high soil-available P conditions. Redundancy analysis showed the significant effects of soil pH, available P in soil, and P content in leaves of *P. pyrifolia* var. *culta* trees on AMF distribution. These results suggested that the accumulation of soil-available P affected AMF communities in the roots of Japanese pear in the orchard environment.

Arbuscular mycorrhizal fungi (AMF) are ubiquitous in most terrestrial plant communities and form mutualistic associations with the majority of plant species (46). This symbiosis confers the host plants with numerous benefits such as growth promotion through improved mineral nutrients, particularly phosphorus (P), and alleviation of diseases (6) and abiotic stresses, namely drought (5), heavy metal toxicity (32), salinity (17), *etc*. AMF symbioses usually display less specificity under pot culture conditions with inoculation of a single fungal strain (46); however, some studies have revealed that AMF communities differ among different plant species in the same ecosystem, which indicates the existence of preferences between plants and AMF (52, 53). The effect of floristic composition on AMF communities was also experimentally demonstrated in a grassland microcosm (24). Furthermore, AMF have functionally diversified to have different effects on plant growth (31, 47). These results suggested the importance of AMF communities in plant ecosystems. Because of their importance for agricultural plants, AMF could be used to minimize the dependence on chemical fertilizers; however, mycorrhizal technology is not much utilized in agricultural practice because of the poor understanding of the basic biology and ecology of AMF in agricultural ecosystems.

Many studies have demonstrated that the AMF colonization rate decreases with the addition of P to the soil (1, 12, 34, 42, 48, 50). The effect of increasing soil-available P on the AMF colonization rate can differ depending on the AMF species. For example, Thomson *et al.* (50) reported that the percentage of root length colonized by *Gigaspora calospora* was decreased by a greater extent than that of *Glomus fasciculatum* with increasing soil-available P. In agricultural systems, some studies have shown that higher levels of inorganic fertilizer input resulted in lower AMF diversity (13, 20, 36). Moreover, it was also suggested that less efficient AMF might be selected with high fertilizer input (25, 45).

Soil-available P in arable land in Japan has been increasing (35). It is often excessive, especially in orchards, because of the repeated application of chemical fertilizers, which may have some detrimental effects on AMF. Ishii *et al.* (22) showed that AMF proliferation was suppressed in citrus orchards with high levels of P. Youpensuk *et al.* (56) also found that AMF root colonization rates and spore densities were significantly decreased in tangerine (*Citrus reticulate*) orchards containing more than 500 mg kg^−1^ of available P in soil.

Pears (genus *Pyrus*, *Rosaceae*) are cultivated for eating throughout the world. Orchard trees of *Rosaceae* are known to be host plants of AMF (10, 11, 39, 40, 41). For *P. commun* L., growth enhancement was shown for seedlings with AMF inoculation (16). Moreover, Lopez *et al.* (30) showed that foliar levels of N, P, and Zn of *P. commun* rootstock were increased by *Glomus intraradices* and *G. mosseae* inoculations under field conditions. *P. pyrifolia* var. *culta* (Japanese pear) is a widely cultivated fruit tree species in Japan; however, there has been little research into AMF for this tree species. In the present study, we examined the colonization rates and communities of AMF in the roots of *P. pyrifolia* var. *culta* in 13 orchards in Tottori Pref., Japan, as well as soil chemical properties to investigate the effect of fertilization on AMF.

## Materials and Methods

### Sampling

In Tottori Prefecture, 13 orchards managed by different farmers were selected as the study sites (Table 1), and sampling was conducted in June and July 2010. For sampling, four trees of *P. pyrifolia* var. *culta* cultivar ‘Osa-Gold’ were randomly selected in each orchard, and three soil core samples (5 cm in diameter and 10 cm in depth) containing roots of Japanese pear were collected from three points at a distance of approximately 70 cm from the tree base. The three soil cores were mixed to prepare one soil sample. Furthermore, 10 leaves were collected from the middle of the spurs for each selected tree.

### Soil chemical analysis

Soil samples collected from the soil cores were dried at room temperature for a week after removing root samples. They were then analyzed for soil chemical properties. The soil pH (H_2_O) at 1:2.5 of the soil–water ratio and the available P (Truog P; 51) were measured. Total C and total N were analyzed using an Elementar varioEL CHNS analyser (Elementar, Hanau, Germany).

### Leaf P content

The collected pear leaves were washed with tap water, rinsed with distilled water, and then dried at 70°C for 48 h. The leaves were ground using an Oster Blender (Osaka Chemical, Osaka, Japan) and the leaf samples were then digested using H_2_SO_4_ and H_2_O_2_ in a heat block at approximately 200°C. Subsequently, leaf P content was determined using the vanadomolybdate spectrophotometric method (8).

### AMF colonization rate

Root samples were carefully washed with tap water to remove attached soil debris, and fine roots were collected. AMF colonization rates were determined using approximately 15 mg fresh fine roots according to the method of Brundrett *et al.* (9) as follows. The fine roots were cleared in 10% KOH at 121°C for 20 min by autoclaving. After sequential rinsing with distilled water, alkaline H_2_O_2_, and 2% HCl, the roots were stained with 0.05% trypan blue at 100°C in a water bath for 10 min. The stained roots were then stored in a lactic acid:glycerol:water (1:1:1) solution. The AMF colonization rate was determined by the gridline intersection method with at least 100 intersections.

### Molecular diversity analysis

DNA was extracted from of the remaining fresh fine roots (*ca.* 20–50 mg) using a DNeasy Plant Mini Kit (Qiagen, Hilden, Germany). A partial sequence (approximately 750 bp) of the AM fungal nuclear small-subunit ribosomal RNA gene (SSU rDNA) was amplified by polymerase chain reaction (PCR) from 1 μL extracted DNA solution using the AMF-specific primers AML1 and AML2 (28) with Takara Ex TaqHot Start Version (Takara Bio, Otsu, Japan). PCR was performed using a Program Temp Control System (Astec, Fukuoka, Japan) in a total volume of 30 μL containing 0.15 μL Taq DNA polymerase, 3 μL PCR buffer (consisting of 100 mM Tris-HCl (pH 8.3), 500 mM KCl and 15 mM MgCl_2_), 200 μM of each deoxynucleotide triphosphate (dNTP), and 0.25 μM of each primer. The PCR conditions were as follows: an initial denaturation step at 94°C for 2 min, followed by 35 cycles of 94°C for 30 s, 50°C for 30 s, and 72°C for 1 min, and a final elongation step at 72°C for 5 min. After purification using the Gel Indicator DNA Extraction Kit (BioDynamics Laboratory, Tokyo, Japan), the PCR products were ligated into pGEM-T Easy Vector System I (Promega, Madison, WI, USA) according to the manufacturer’s instructions. They were then transformed into competent cells (Competent high DH5α; Toyobo, Osaka, Japan). For each sample, at least 16 cloned products (white colonies) were randomly selected, and the plasmid DNA containing the PCR product was extracted from 1.5 mL cultured competent cells using the Mag Extractor Plasmid (Toyobo, Osaka, Japan). The DNA inserts were sequenced using a BigDye Terminator v3.1 Cycle Sequencing Kit (Applied Biosystems, Carlsbad, CA. USA) and the promoter primers T7 and SP6 on a 3130 Genetic Analyzer (Hitachi, Tokyo, Japan). For all sequenced data, multiple sequence alignments were performed using ClustalX version 2.0.12 (27). The aligned sequences were analyzed by the neighbor-joining method (43) with bootstrap analyses of 1,000 replications (15). The phylogenetic trees were drawn using TreeView (38). AMF phylotypes were defined on the basis of tree topology and sequence similarity computed by ClustalX. A rarefaction curve was computed for each sample by plotting the number of AMF phylotypes detected against the number of sequences using Analytic Rarefaction version 1.3 (https://www.uga.edu/_strata/software/AnRare/Readme.html). For each sample, additional clones were sequenced until the rarefaction curve tended to plateau.

Some representative DNA sequences were arbitrarily selected for each AMF phylotype and deposited into the DNA Data Bank of Japan (DDBJ) database with accession numbers AB694978–AB695066. The selected sequences were subjected to BLAST searches (3), and the similar sequences were downloaded from the GenBank database. Multiple sequence alignments, neighbor-joining analysis, and phylogenetic tree drawing were performed as described above for the sequenced and downloaded data.

### Data analysis

The relationships between the soil chemical properties or leaf P and AMF colonization rates or number of AMF phylotypes were investigated by Pearson’s correlation coefficient test.

To examine the relationship between the AMF distribution and the environmental factors, multivariate analyses were performed using CANOCO 4.5 (49). In the data table of response variables, *i.e.*, the distribution of AMF phylotypes, the presence or absence of each phylotype was scored as so-called dummy variables (“1” and “0” for the presence and absence, respectively) in each sample. For the data of explanatory (environmental) variables, soil-available P, soil pH, soil total C, soil total N, and leaf P content were used. First, detrended correspondence analysis (DCA) was performed for the response variable data to estimate the heterogeneity through the length of the community composition gradients in species turnover units. DCA was performed with detrending by segments. After confirming the length of the community composition gradients on the first DCA axis, principal component analysis (PCA) was performed to infer the relationship between AMF distribution and the environmental variables. PCA was performed with scaling on interspecies correlations with division by standard deviation and centering by species. The resulting diagram was displayed using CanoDraw. To evaluate the effects of environmental variables, redundancy analysis (RDA) was then applied with scaling on interspecies correlations with division by standard deviation and centering by species, in which Monte Carlo permutation tests with unrestricted 999 permutations were performed for manually selected environmental variables.

## Results

### Soil analysis and AMF colonization rates

**Fig. 1:**
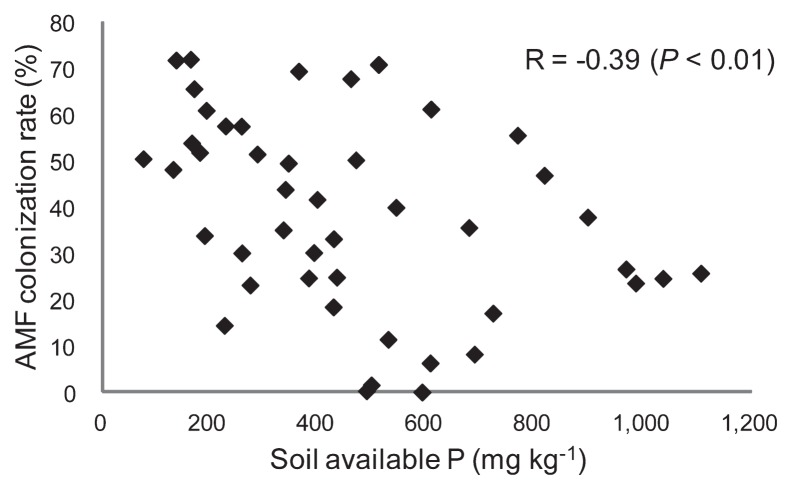
Correlation between soil-available phosphorus (P) and the colonization rate of arbuscular mycorrhizal fungi (AMF) in the roots of *Pyrus pyrifolia* var. *culta* (Japanese pear).

The soil type of the examined area was basically andosol caused by volcanic ash; however, the soil color was not blackish in most examined orchards because of the disappearance of topsoil during the preparation of orchards. The soil chemical properties and AMF colonization rates are shown in Table 2. The soil-available P was found to vary markedly, 75.7 to 1,200 mg kg^−1^. The AMF colonization rate was determined for 45 samples having sufficient fine roots for examination. The rate varied from 0.2 to 71.9%, which was negatively correlated with the amount of soil-available P with a coefficient of −0.39 (*P* <0.01) (Fig. 1). For other soil chemical properties and leaf P, significant correlations were not found in the relationships with AMF colonization rates (Table S1).

### Molecular analysis on AMF community

**Fig. 2:**
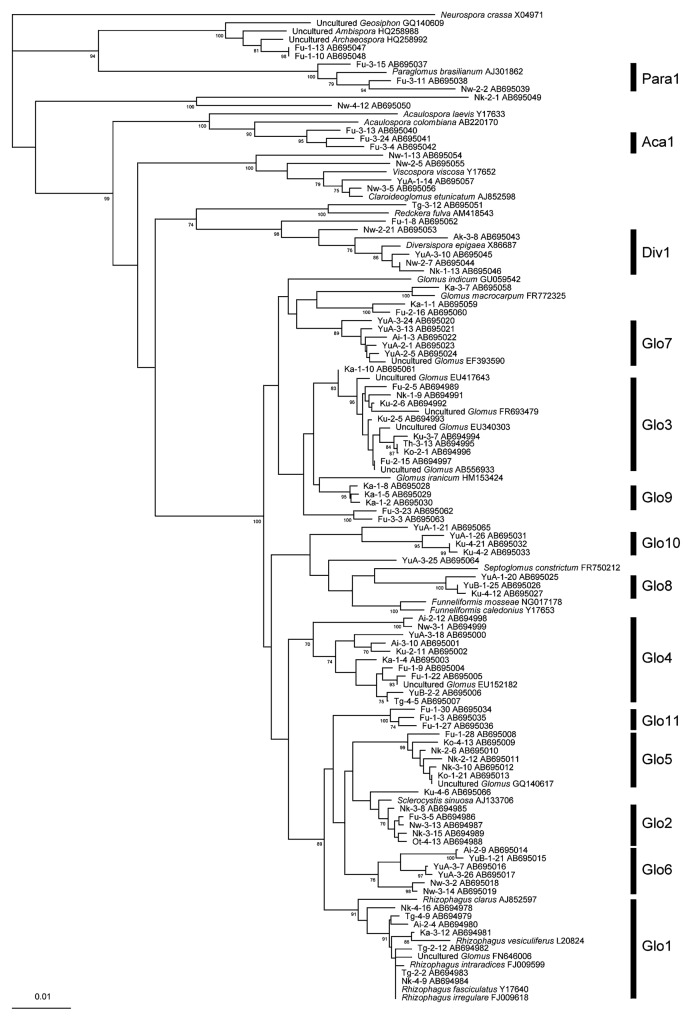
A neighbor-joining phylogenetic tree based on partial sequences of SSU rDNA of arbuscular mycorrhizal fungi (AMF) in the roots of *Pyrus pyrifolia* var. *culta* (Japanese pear) and in the GenBank database. The tree is rooted to *Neurospora crassa* (X04971) in Ascomycota. The sequence numbers are related to the orchard, tree, and clone numbers. The division of phylotypes (Glo1–Glo11, Aca1, Div1, and Para1) is shown. Bootstrap values are shown when they exceed 70% (1,000 replications). The scale is shown so that evolutionary distances can be inferred. Accession numbers are given for all sequences.

**Fig. 3:**
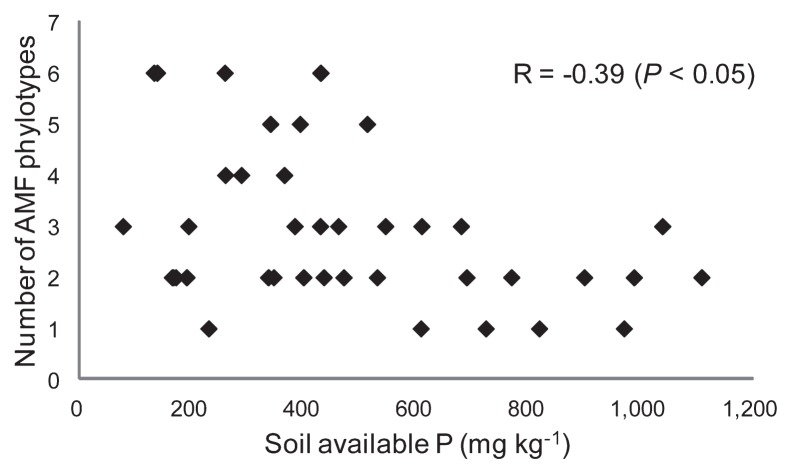
Correlation between soil-available phosphorus (P) and the number of phylotypes of arbuscular mycorrhizal fungi (AMF) in the roots of *Pyrus pyrifolia* var. *culta* (Japanese pear).

**Fig. 4:**
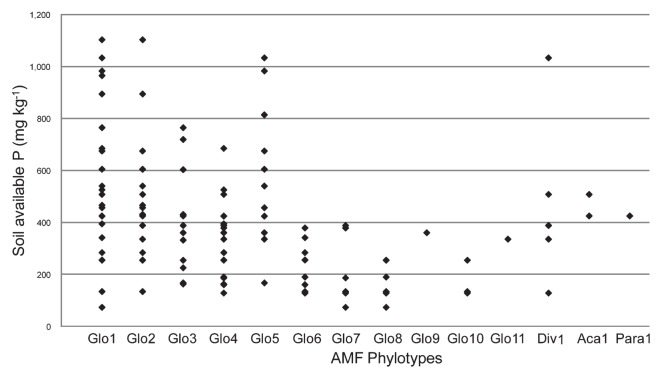
Relationship between soil-available phosphorus (P) and the distribution of phylotypes of arbuscular mycorrhizal fungi (AMF) in the roots of *Pyrus pyrifolia* var. *culta* (Japanese pear).

For 39 samples having sufficient fine roots for examination (*ca.* 20–50 mg), molecular analysis was performed to examine the AMF communities. For each sample, 16–36 clones were sequenced. After excluding putative chimera sequences, 618 sequences were obtained. All of the obtained sequences were divided into 14 phylotypes by neighbor-joining phylogenetic analysis so that each phylotype had sequence similarity of more than 96%. Each phylotype was determined to include at least 3 sequences obtained. Eleven of the phylotypes were *Glomus*, and one each was *Paraglomus*, *Acaulospora*, and *Diversispora*. All phylotypes were supported by bootstrap values of more than 70%. For the taxonomy of AMF, new revised genera based on the molecular phylogeny were proposed (26, 44), and the new genera were shown for the identified AMF species in the phylogenetic tree (Fig. 2); however, allocations to the new genera were difficult for most of the phylotypes in this study because of the lack of related identified species. Thus, we used the former taxonomy based on the spore morphologies to express the phylotypes. The phylogenetic trees constructed using selected and downloaded DNA sequences are shown in Fig. 2. Only two phylotypes were affiliated with morphologically identified AMF species: Glo1 with *G. intraradices* (*Rhizophagus intraradices*) and Div1 with *Diversispora epigaea*. The number of AMF phylotypes was negatively correlated with soil-available P with a coefficient of −0.39 (*P* <0.05) (Fig. 3). Investigation of the relationship between the distribution of AMF phylotypes and the available P showed that only four AMF phylotypes, *i.e.*, Glo1, Glo2 Glo5, and Div1, were detected in the soil with more than 800 mg kg^−1^ of available P (Fig. 4). For other soil chemical properties and leaf P, significant correlations were not found in the relationships with the number of AMF phylotypes (Table S1).

### AMF community and environmental variables

**Fig. 5:**
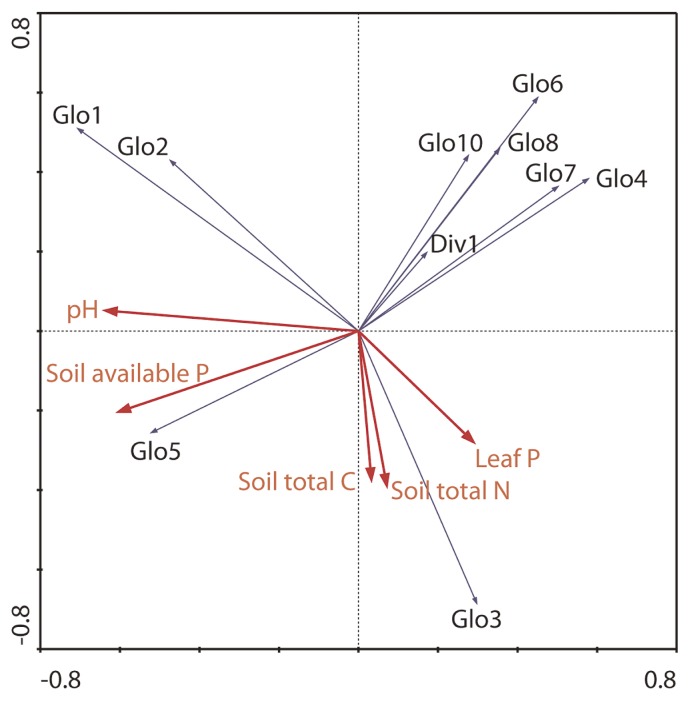
Diagram of principal component analysis (PCA) on communities of arbuscular mycorrhizal fungi (AMF) with environmental variables, soil-available P, soil pH, soil total nitrogen (N), soil total carbon (C), and P content in the leaves (leaf P), in the examined orchards of *Pyrus pyrifolia* var. *culta* (Japanese pear). The eigenvalues of the first and second PCA axes were 0.242 and 0.214, respectively.

From DCA for the response variable data, the length of the community composition gradients of the first axis was computed to be 3.63. From this result, we used PCA as the linear ordination method. The resulting ordination is presented in Fig. 5. The eigenvalues of the first and second axes were 0.242 and 0.214, respectively. The cumulative percentage variance of species data showed that the first two PCA axes explain 45.7% of the variability in species data. PCA on AMF distribution and environmental variables suggested that many AMF prefer soil conditions with lower available soil P. The relative preference for higher soil-available P in Glo1, Glo2, and Glo5 AMF was also suggested in PCA. The results of Monte Carlo permutation tests on RDA indicated that environmental variables, such as soil pH, soil-available P, and leaf P, affected the AMF distribution significantly (*P* <0.05), in which the effect of soil available P was stronger than that of leaf P (Table 3). These results suggested that soil P accumulation caused by fertilization could be an influential factor on AMF communities in the examined orchards.

## Discussion

Most AMF detected in the roots of Japanese pear in this study were *Glomus* spp., although *Paraglomus*, *Acaulospora*, and *Diversispora* spp. were also detected (Fig. 2). Lee *et al.* (28) reported that the PCR primer set AML1 and AML2 used in this study can amplify the sequences of most AMF, and their adaptation for diverse AMF taxa was confirmed in this study; therefore, the dominant detection of *Glomus* fungi in the roots of Japanese pear can be a reflection of the actual AMF communities.

Most DNA sequences obtained in this study were not closely related to those of any morphologically identified AMF species in the GenBank database. Other studies also revealed that most AMF in tree roots are not closely related to the identified AMF species (7, 19, 21, 55). For *Rosaceae* trees, Wubet *et al.* (54) investigated the molecular diversity of AMF in *Prunus africana* in the dry afromontane forests of Ethiopia. In phylogenetic analysis, they detected 21 phylotypes, most of which were not assigned to known AMF species. These results may indicate that AMF colonizing tree roots are not inclined to produce conspicuous spores.

The AMF sequences were divided into 14 phylotypes (Fig. 2). Significant negative correlations were found between the soil-available P and the AMF colonization rate or the number of AMF phylotypes (AMF species richness) (Figs. 1 and 3). This result is consistent with some previous studies (2, 14, 29). The figures suggested that the effect of soil-available P on the AMF colonization rate or the number of AMF phylotypes became obvious at certain levels, *e.g.*, 800 mg kg^−1^ for AMF rate and 500 mg kg^−1^ for AMF phylotypes, respectively. Meanwhile, the correlations between leaf P and AMF rate or AMF species richness were not significant. RDA on the AMF distribution and environmental variables showed that soil pH, soil-available P, and leaf P had significant relationships with the AMF distribution in the roots of Japanese pears, in which the effect of soil-available P was stronger than that of leaf P (Table 3). The detrimental effects of soil-available P on AMF colonization have been believed to be caused by changes in the P status of the plant (23, 33); however, examination of the relationships between the P status of trees and the AMF colonization at local roots is difficult under field conditions, especially in orchards. Leaf P can reflect overall P absorption by roots and AMF, and the absorption area includes less fertilized areas and deeper zones; however, the roots in these areas were not evaluated for AMF in this study. It is probable that AMF colonization can be controlled by the P status of each local root through local reaction for enhanced P flux rather than the P status of the whole plant (18), and this may be the reason for the stronger effect of soil P on AMF than leaf P in this study. Soil pH was another factor that strongly affected AMF distribution. An *et al.* (4) also showed that soil pH could be a driving force for AMF communities in comparisons of those in acid sulfate soils and a non-acidic sandy soil; however, the soil pH of the examined orchards in this study was determined by various factors, such as original soil pH, amount of lime and/or magnesia, and fertilizers applied. Because these factors themselves may also affect AMF communities, we refrained from discussing the effect of pH alone. The effects of soil total C and soil total N were smaller than those of soil-available P and soil pH (Table 3).

In Japan, the recommended level of available P in orchard soil is 44–131 mg kg^−1^ according to the Ministry of Agriculture, Forestry, and Fisheries (http://www.maff.go.jp/j/seisan/kankyo/hozen_type/h_dozyo/pdf/chi4.pdf); however, almost all soil samples examined in this study had much higher available P content. Some AMF phylotypes were found to be dominant under higher soil-available P conditions. Glo1 is one such AMF phylotype, and this AMF phylotype includes *G. intraradices*. This AMF species is known as a generalist because it has been found in various environments such as tropical forests, grasslands, temperate deciduous forests, and arable fields (37). Increased abundance of *G. intraradices* in response to fertilization was also found by Johnson (25), who also showed that fertilization changed the composition of AMF communities in field plots, in which AMF from fertilized soil was found to have reduced effects on plant growth. Scullion *et al.* (45) also reported that AMF inocula from organic farms were more effective than those from conventional farms with high fertilizer input. These studies suggested that intensive fertilization may select some AMF with inferior effects on host plants.

The negative correlations between soil-available P and the AMF colonization rate or AMF species richness shown in this study suggested that agricultural practices with excessive fertilization could negate the advantages of AMF. In order to utilize the positive functions of AMF symbiosis in orchards, appropriate application amounts of fertilizer should be further studied with consideration of AMF.

## Supplementary Material



## Figures and Tables

**Table 1:** Location of the study sites

Study site	Latitude	Longitude
Aimi (Ai)	35°20.9′ N	133°24.2′ E
Nawa (Nw)	35°30.0′ N	133°30.5′ E
Nakayama (Nk)	35°30.7′ N	133°33.9′ E
Akasaki (Ak)	35°27.8′ N	133°38.3′ E
Tohaku (Th)	35°29.3′ N	133°39.2′ E
Kurayoshi (Ku)	35°23.4′ N	133°44.7′ E
Togo (Tg)	35°28.0′ N	133°55.1′ E
Yura (YuA, YuB)*	35°28.5′ N	133°44.5′ E
Otsuka (Ot)	35°29.0′ N	134°09.1′ E
Kawahara (Ka)	35°23.5′ N	134°11.2′ E
Koge (Ko)	35°24.5′ N	134°19.9′ E
Fukube (Fu)	35°32.4′ N	134°15.4′ E

* Two different fertilized conditions (A, B) were included in ‘Yura’. ‘A’ was not fertilized, and ‘B’ had conventional fertilization.

**Table 2:** Soil chemical properties, leaf P, AMF colonization rate, and number of phylotypes in each sample

Sample		Soil pH	Soil available P (mg kg^−1^)	Soil total N (g kg^−1^)	Soil total C (g kg^−1^)	Leaf P (mg g^−1^)	AMF colonization rate (%)	Number of AMF phylotypes
Aimi (Ai)	1	5.7	381.2	3.40	42.0	2.7	24.9	3
	2	5.3	257.8	3.82	62.1	2.0	30.3	4
	3	5.5	426.7	4.34	67.9	1.9	18.6	3
	4	6.1	497.0	3.93	52.1	1.8	1.8	—
Nawa (Nw)	1	6.3	468.5	2.97	36.5	2.2	50.4	2
	2	6.1	510.3	3.12	36.6	2.1	71.0	5
	3	5.7	286.3	3.08	41.4	1.8	51.6	4
	4	6.4	459.0	3.76	47.0	2.0	67.9	3
Nakayama (Nk)	1	6.3	390.7	5.37	83.4	2.9	30.3	5
	2	6.5	542.5	5.15	80.8	2.7	40.1	3
	3	6.4	677.3	5.66	91.1	2.4	35.8	3
	4	6.5	607.1	4.78	66.7	2.6	61.3	3
Akasaki (Ak)	1	7.0	967.4	4.87	51.2	2.2	26.8	1
	2	7.0	745.7	4.31	41.7	1.8	—	—
	3	7.0	1,036	5.11	63.2	2.2	24.8	3
	4	7.0	1,136	3.95	41.0	2.3	—	—
Tohaku (Th)	1	4.8	590.6	6.80	83.5	2.0	0.2	—
	2	4.1	488.1	5.62	74.3	2.0	0.5	—
	3	4.4	605.8	6.05	76.4	2.3	6.5	1
	4	5.0	721.6	4.95	58.9	2.2	17.3	1
Kurayoshi (Ku)	1	5.2	227.9	7.72	129.0	2.6	57.7	1
	2	5.3	165.2	7.40	122.5	2.7	54.0	2
	3	5.5	169.6	6.98	111.4	2.4	65.7	2
	4	5.6	257.0	6.31	95.5	2.4	57.6	6
Togo (Tg)	1	5.3	882.2	2.77	32.0	2.6	—	—
	2	5.3	527.9	2.07	24.9	2.3	11.6	2
	3	5.8	687.4	2.21	26.7	2.3	8.4	2
	4	5.3	396.9	2.09	26.4	2.5	41.8	2
Yura A (YuA)	1	6.1	136.4	3.81	44.0	2.7	71.9	6
	2	6.2	75.7	3.04	34.9	2.1	50.6	3
	3	5.3	130.7	4.17	43.0	2.4	48.3	6
	4	5.3	188.9	4.03	45.3	2.4	34.0	2
Yura B (YuB)	1	4.6	192.1	4.03	44.6	2.3	61.1	3
	2	5.2	163.0	2.99	35.1	2.6	72.1	2
	3	4.6	273.1	3.53	38.0	2.5	23.3	—
	4	5.0	225.7	2.56	29.2	2.5	14.6	—
Otsuka (Ot)	1	6.6	1,106	4.28	48.1	1.9	25.9	2
	2	6.7	959.9	4.03	48.3	2.2	—	—
	3	6.6	1,137	4.64	55.4	2.1	—	—
	4	6.4	896.7	3.58	42.5	2.2	38.0	2
Kawahara (Ka)	1	5.2	362.8	2.75	39.9	2.1	69.5	4
	2	4.9	227.5	2.65	39.8	1.9	—	—
	3	4.8	343.8	3.19	47.2	1.8	49.6	2
	4	4.8	180.1	2.70	40.8	2.1	52.0	—
Koge (Ko)	1	6.6	985.2	2.87	31.1	2.4	23.8	2
	2	6.5	767.1	2.58	27.8	2.7	55.7	2
	3	6.8	1,200	3.19	34.8	2.1	—	—
	4	6.5	816.5	2.80	31.1	2.5	47.0	1
Fukube (Fu)	1	5.7	338.1	3.95	49.3	2.5	44.0	5
	2	6.4	334.3	4.77	60.8	2.7	35.2	2
	3	6.4	427.3	2.78	35.0	2.5	33.3	6
	4	6.2	433.0	3.04	34.4	2.7	25.0	2

**Table 3:** Result of Monte Carlo permutation tests (999 permutations) from redundancy analysis (RDA) of the effect of environmental variables on arbuscular mycorrhizal fungal community

Environmental variables	*F* value	*P* value
Soil pH	4.69	0.001
Soil vailable P	4.45	0.001
Leaf P	2.27	0.032
Soil total N	2.04	0.054
Soil total C	1.78	0.072
