# Supplementary material for: Communities of Arbuscular Mycorrhizal Fungi in the Roots of Pyrus pyrifolia var. culta (Japanese Pear) in Orchards with Variable Amounts of Soil-Available Phosphorus
Source: Microbes Environ. 2012 Dec 19;28(1):105–11. doi: 10.1264/jsme2.ME12118 (PMC4070685; doi:10.1264/jsme2.ME12118)
Supplement: Supplementary file 1 [file 28_105_s1.pdf]

Table S1 Pearson's correlation coefficients between AMF colonization rate or the number of phylotypes and soil pH, total N, total C or leaf P

	Soil pH	Soil total N	Soil total C	Leaf P
AMF colonization rate	0.16 (0.305*)	-0.04 (0.772)	0.04 (0.771)	0.21 (0.174)
The number of phylotypes	0.07 (0.681)	-0.07 (0.677)	-0.04 (0.810)	0.08 (0.630)

**P* value
